# Supplementary material for: One-Year Outcomes after Surgical versus Transcatheter Aortic Valve Replacement with Newer Generation Devices
Source: J Clin Med. 2021 Aug 20;10(16):3703. doi: 10.3390/jcm10163703 (PMC8396948; doi:10.3390/jcm10163703)
Supplement: Supplementary file 1 [file jcm-10-03703-s001.zip › jcm-1324221-supplementary.pdf]

Supplemental Material

One-year Outcome after Surgical versus Transcatheter Aortic Valve Replacement with Newer Generation Devices

Rosato S, et al.

Content

Supplemental Table S1. STROBE Statement—checklist of items that should be included in reports of observational studies

Supplemental Table S2 Types of transcatheter aortic valve replacement devices in the overall and propensity score matched groups.

Supplemental Table S3. Access sites for transcatheter aortic valve in the overall and propensity score matched groups.

Supplemental Figure S1. Propensity scores distribution before (in blue) and after propensity score matching (in red).

Supplemental Figure S2. Balance of covariates between the study groups before (in blue) and after propensity score matching (in red).

List of the OBSERVANT I and II investigators

Supplemental Table S1. STROBE Statement—checklist of items that should be included in reports of observational studies

	Item No	Recommendation	Page
**Title and abstract**	1	(*a*) Indicate the study's design with a commonly used term in the title or the abstract	1
		(*b*) Provide in the abstract an informative and balanced summary of what was done and what was found	1
**Introduction**			
Background/rationale	2	Explain the scientific background and rationale for the investigation being reported	4
Objectives	3	State specific objectives, including any prespecified hypotheses	4
**Methods**			
Study design	4	Present key elements of study design early in the paper	4-6
Setting	5	Describe the setting, locations, and relevant dates, including periods of recruitment, exposure, follow-up, and data collection	4-6
Participants	6	(*a*) *Cohort study*—Give the eligibility criteria, and the sources and methods of selection of participants. Describe methods of follow-up<br><br>*Case-control study*—Give the eligibility criteria, and the sources and methods of case ascertainment and control selection. Give the rationale for the choice of cases and controls	4-6

		Cross-sectional study—Give the eligibility criteria, and the sources and methods of selection of participants	
		(*b*) *Cohort study*—For matched studies, give matching criteria and number of exposed and unexposed<br>*Case-control study*—For matched studies, give matching criteria and the number of controls per case	7
Variables	7	Clearly define all outcomes, exposures, predictors, potential confounders, and effect modifiers. Give diagnostic criteria, if applicable	6
Data sources/ measurement	8*	For each variable of interest, give sources of data and details of methods of assessment (measurement). Describe comparability of assessment methods if there is more than one group	6
Bias	9	Describe any efforts to address potential sources of bias	7
Study size	10	Explain how the study size was arrived at	-
Quantitative variables	11	Explain how quantitative variables were handled in the analyses. If applicable, describe which groupings were chosen and why	7
Statistical methods	12	(*a*) Describe all statistical methods, including those used to control for confounding	7
		(*b*) Describe any methods used to examine subgroups and interactions	7
		(*c*) Explain how missing data were addressed	7
		(*d*) *Cohort study*—If applicable, explain how loss to follow-up was addressed<br>*Case-control study*—If applicable, explain how matching of cases and controls was addressed<br>*Cross-sectional study*—If applicable, describe analytical methods taking account of sampling strategy	5
		(*e*) Describe any sensitivity analyses	7

**Results**			**Page**
Participants	13*	(a) Report numbers of individuals at each stage of study—eg numbers potentially eligible, examined for eligibility, confirmed eligible, included in the study, completing follow-up, and analysed	8, Fig. 1
		(b) Give reasons for non-participation at each stage	8, Fig. 1
		(c) Consider use of a flow diagram	Fig. 1
Descriptive data	14*	(a) Give characteristics of study participants (eg demographic, clinical, social) and information on exposures and potential confounders	8, Fig. 1
		(b) Indicate number of participants with missing data for each variable of interest	8, Fig. 1
		(c) *Cohort study*—Summarise follow-up time (eg, average and total amount)	8
Outcome data	15*	*Cohort study*—Report numbers of outcome events or summary measures over time	8,9
		Case-control study—Report numbers in each exposure category, or summary measures of exposure	
		Cross-sectional study—Report numbers of outcome events or summary measures	
Main results	16	(*a*) Give unadjusted estimates and, if applicable, confounder-adjusted estimates and their precision (eg, 95% confidence interval). Make clear which confounders were adjusted for and why they were included	8,9

		(*b*) Report category boundaries when continuous variables were categorized	8,9
		(*c*) If relevant, consider translating estimates of relative risk into absolute risk for a meaningful time period	
Other analyses	17	Report other analyses done—eg analyses of subgroups and interactions, and sensitivity analyses	8,9
**Discussion**			
Key results	18	Summarise key results with reference to study objectives	10
Limitations	19	Discuss limitations of the study, taking into account sources of potential bias or imprecision. Discuss both direction and magnitude of any potential bias	12
Interpretation	20	Give a cautious overall interpretation of results considering objectives, limitations, multiplicity of analyses, results from similar studies, and other relevant evidence	12
Generalisability	21	Discuss the generalisability (external validity) of the study results	10
**Other information**			
Funding	22	Give the source of funding and the role of the funders for the present study and, if applicable, for the original study on which the present article is based	1

Supplemental Table S2. Types of transcatheter aortic valve replacement devices in the overall and propensity score matched groups.

Devices and their size	Before propensity score matching n = 2520	After propensity score matching n = 1008
Acurate L	78 (3.1)	34 (3.4)
Acurate M	121 (4.8)	58 (5.8)
Acurate S	70 (2.8)	33 (3.3)
Engager 23 mm	2 (0.1)	1 (0.1)
Engager 26 mm	5 (0.2)	3 (0.3)
Evolut Pro 23 mm	21 (0.8)	7 (0.7)
Evolut Pro 26 mm	130 (5.2)	52 (5.2)
Evolut Pro 29 mm	167 (6.6)	56 (5.6)
Evolut R 23 mm	87 (3.5)	26 (2.6)
Evolut R 26 mm	340 (13.5)	143 (14.2)
Evolut R 29 mm	349 (13.7)	135 (13.4)
Evolut R 34 mm	245 (9.7)	89 (8.8)
Lotus 23 mm	4 (0.2)	2 (0.2)
Lotus 25 mm	5 (0.2)	3 (0.3)
Lotus 27 mm	1 (0.1)	0

Lotus 29 mm	1 (0.1)	0
Portico 23 mm	26 (1.0)	5 (0.5)
Portico 25 mm	67 (2.7)	29 (2.9)
Portico 27 mm	54 (2.1)	21 (2.1)
Portico 29 mm	44 (1.8)	20 (2.0)
Sapien 3 20 mm	16 (0.6)	4 (0.4)
Sapien 3 23 mm	283 (11.2)	116 (11.5)
Sapien 3 26 mm	282 (11.2)	130 (12.9)
Sapien 3 29 mm	114 (4.5)	41 (4.1)
Not reported	8 (0.3)	0

Data is reported as counts and percentages (in parentheses).

Supplemental Table S3. Access sites for transcatheter aortic valve replacement in the overall and propensity score matched groups.

Access sites	Before propensity score matching n = 2520	After propensity score matching n = 1008
Transfemoral	2326 (92.3)	939 (93.2)
Transaxyllary/subclavian	94 (3.7)	32 (3.2)
Transapical	79 (3.1)	32 (3.2)
Other access sites	18 (0.7)	5 (0.5)
Not reported	3 (0.1)	0

Data is reported as counts and percentages (in parentheses).

(in red).

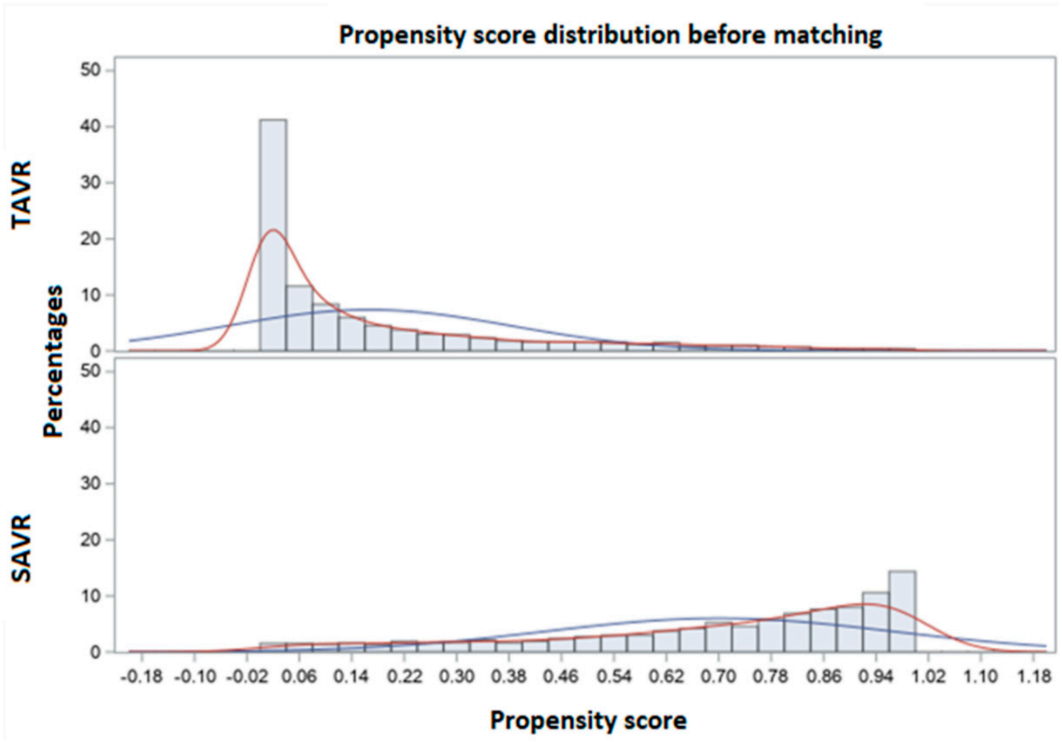

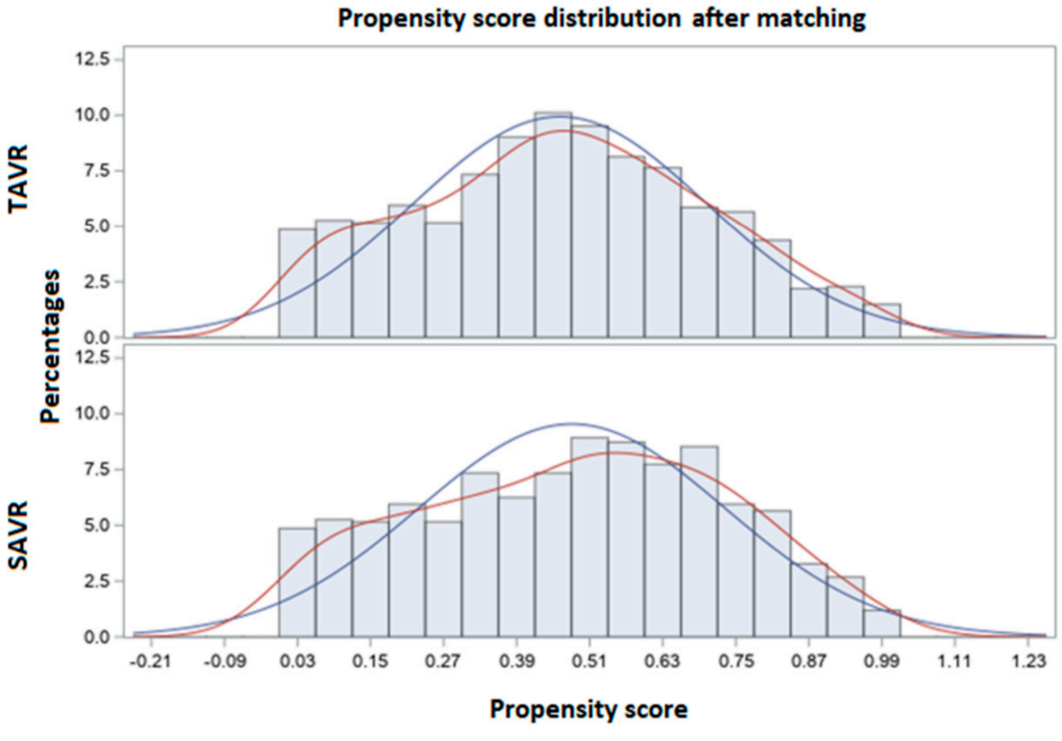

Supplemental Figure S1. Propensity scores distribution before (in blue) and after propensity score matching (in red).

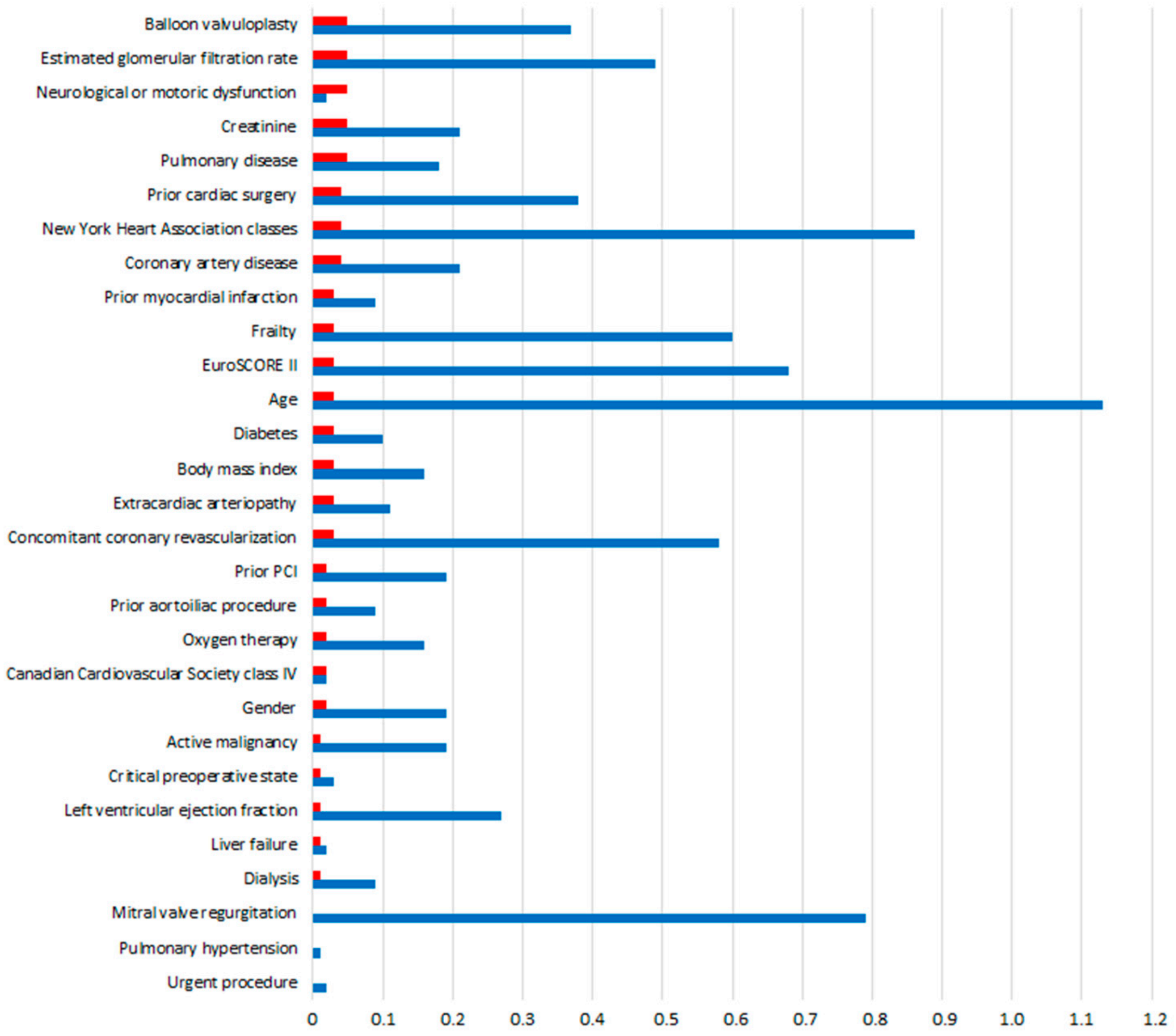

Supplemental Figure S2. Balance of covariates between the study groups before (in blue) and after propensity score matching (in red).

OBSERVANT I Investigators:

Fulvia Seccareccia, Paola D'Errigo, Stefano Rosato, Alice Maraschini, Gabriella Badoni, National Centre for Epidemiology, Surveillance and Health Promotion - ISS;

Corrado Tamburino, Marco Barbanti, SICI-GISE;

Gennaro Santoro, FIC, ANMCO;

Francesco Santini, Francesco Onorati, Claudio Grossi, SICCH;

Marco Ranucci, Remo Daniel Covello, ITACTA;

Danilo Fusco, Epidemiology Dept. Lazio Region;

Rossana De Palma, Emilia Romagna Region;

Salvatore Scondotto, Sicilia Region.

Participating cardiology centers

1. Città della Salute e della Scienza - A.O.U. Molinette - San Giovanni Battista di Torino, Torino. Marra S., Marra S., D'Amico M.
2. Città della Salute e della Scienza - A.O.U. Molinette - San Giovanni Battista di Torino, Torino. Gaita F., Moretti C.
3. Ospedale Mauriziano "Umberto I", Torino. De Benedictis M., Aranzulla T.
4. A.O. Nazionale Ss. Antonio e Biagio e Cesare Arrigo, Alessandria. Pistis G., Reale M.
5. Istituto Clinico S.Ambrogio, Milano. Bedogni F., Brambilla N.
6. Fondazione IRCSS Policlinico San Matteo, Pavia. Ferrario M., Ferrero L., Vicinelli P.
7. Fondazione San Raffaele del Monte Tabor, Milano. Colombo A., Chieffo A., Ferrari A.
8. I.R.C.C.S. Policlinico San Donato, San Donato M.se (MI). Inglese L., Casilli F.
9. Spedali Civili di Brescia - Università, Brescia. Ettori F., Frontini M.
10. Ospedale Luigi Sacco - A.O. - Polo Universitario, Milano. Antona C., Piccaluga E.
11. A.O. Ospedale Niguarda Cà Granda, Milano. Klugmann S., De Marco F.
12. A.O. Bolognini Seriate, Seriate (BG). Tespili M., Saino A.
13. Fondazione Poliambulanza Istituto Ospedaliero, Brescia. Leonzi Ornella, Rizzi Andrea
14. Ospedale "S. Maria di Ca' Foncello", Treviso. Franceschini Grisolia E., Franceschini Grisolia E.
15. A.O. di Padova, Padova. Isabella G., Fraccaro C.
16. A.O.U. Santa Maria Della Misericordia di Udine, Udine. Proclemer A., Bisceglia T., Armellini I.
17. A.O.U. San Martino, Genova. Vischi M., Parodi E.
18. A.O.U. di Parma , Parma. Vignali L., Ardissimo D.
19. Policlinico S Orsola Malpighi , Bologna. Marzocchi A., Marrozzini C.
20. Maria Cecilia Hospital , Ravenna. Cremonesi A., Colombo F.
21. A.O.U. Pisana, Pisa. Petronio S., Giannini C.
22. A.O.U. Senese Le Scotte, Siena. Pierli C., Iadanza A.
23. A.O.U. Careggi, Firenze. Santoro G., Meucci F.
24. Ospedale del Cuore Fondazione CNR Regione Toscana G. Monasterio, Massa. Berti S., Mariani M.
25. European Hospital, Roma. Tomai F., Ghini A.
26. A.O. S Camillo-Forlanini , Roma. Violini R., Confessore P.
27. Policlinico Gemelli Cardiologia, Roma. Crea F., Giubilato S.
28. Policlinico Umberto I, Roma. Sardella G., Mancone M.
29. A.O.U. Integrata Verona, Verona. Ribichini F., Vassanelli C., Dandale R.

30. A.O.OO.RR.S. Giovanni di Dio e Ruggi d`Aragona - A.O.U. di Salerno, Salerno. Giudice P., Vigorito F.

31. Casa Di Cura Città Di Lecce Srl , Lecce. Liso A., Specchia L.

32. A.O.U. Mater Domini, Catanzaro. Indolfi C., Spaccarotella C.

33. A.R.N.A.S. Ospedale Civico - Di Cristina - Benfratelli, Palermo. Stabile A., Gandolfo C

34. A.O.U. "Policlinico - Vittorio Emanuele" - Ospedale Ferrarotto, Catania. Tamburino C., Ussia G.

Participating cardiac surgery centers

1. Villa Maria Pia Hospital Gruppo Villa Maria Cardiochirurgia, Torino. Comoglio C., Dyrda O.

2. Città della Salute e della Scienza - A.O.U. Molinette - San Giovanni Battista di Torino, Torino. Rinaldi M., Salizzoni S.

3. Azienda Ospedaliera Universitaria Maggiore della Carità, Novara. Micalizzi E.

4. A.O. S. Croce e Carle, Cuneo. Grossi C., Di Gregorio O.

5. A.O. Nazionale Ss. Antonio e Biagio e Cesare Arrigo, Alessandria. Scoti P., Costa R.

6. Ospedale Mauriziano "Umberto I", Torino. Casabona R., Del Ponte S.

7. Istituto Clinico S.Ambrogio, Milano. Panisi P., Spira G.

8. Fondazione Poliambulanza Istituto Ospedaliero, Brescia. Troise G., Messina A.

9. Fondazione I.R.C.S.S. Policlinico San Matteo, Pavia. Viganò M., Aiello M.

10. Fondazione San Raffaele del Monte Tabor, Milano. Alfieri O., Denti P.

11. I.R.C.C.S. Policlinico San Donato, San Donato M.se (MI). Menicanti L., Agnelli B.

12. IRCCS Multimedica , Milano. Donatelli F.

13. Spedali Civili di Brescia - Università, Brescia. Muneretto C., Frontini M.

14. Spedali Civili di Brescia - Università, Brescia. Rambaldini M., Frontini M.

15. A.O. Ospedale di Lecco - Presidio Alessandro Manzoni, Lecco. Gamba A., Tasca G.

16. Ospedali Riuniti di Bergamo - A.O., Bergamo. Ferrazzi P., Terzi A.

17. Ospedale Luigi Sacco - A.O. - Polo Universitario, Milano. Antona C., Gelpi G.

18. A.O. Ospedale Niguarda Cà Granda, Milano. Martinelli L., Bruschi G.

19. Presidio Ospedaliero S.Chiara - Ospedale di Trento, Trento. Graffigna A.C.

20. A.O.U. Integrata Verona, Verona. Mazzucco A.

21. A.O.U. Ospedali Riuniti di Trieste - Ospedale di Cattinara, Trieste. Pappalardo A., Gatti G.

22. A.O.U. Santa Maria Della Misericordia di Udine, Udine. Livi U., Pompei E.

23. ICLAS - Istituto Clinico Ligure di Alta Specialità , Rapallo (GE). Coppola R., Gucciardo M.

24. A.O.U. San Martino, Genova. Passerone G., Parodi E.

25. Salus Hospital spa, Reggio Emilia. Albertini A., Caprili L.

26. Hesperia Hospital Modena S.r.l. , Modena. Ghidoni I., Gabbieri D.

27. Maria Cecilia Hospital, Ravenna. La Marra M., Aquino T.

28. Azienda Ospedaliero - Universitaria di Parma, Parma. Gherli T.

29. Policlinico S. Orsola Malpighi , Bologna. Di Bartolomeo R., Savini C.

30. Villa Maria Beatrice Hospital , Firenze. Popoff G., Innocenti D.

31. A.O.U. Pisana, Pisa. Bortolotti U., Pratali S.

32. A.O.U. Careggi, Firenze. Stefano P., Blanzola C.

33. Ospedale del Cuore Fondazione CNR Regione Toscana G. Monasterio, Massa. Glauber M., Cerillo A., Chiaramonti F.

34. A.O. Santa Maria, Terni. Pardini A., Fioriello F.

35. A.O. G. M. Lancisi, Ancona. Torracca L., Rescigno G.

36. European Hospital, Roma. De Paulis R., Nardella S.

37. A.O. S Camillo-Forlanini, Roma. Musumeci F., Luzi G.

38. Policlinico Gemelli, Roma. Possati G., Bonalumi G.

39. Università Campus Bio-Medico di Roma, Roma. Covino E., Pollari F.

40. A.O. Sant'Andrea, Roma. Sinatra R., Roscitano A.

41. Policlinico Tor Vergata, Roma. Chiariello L., Nardi P.

42. Clinica San Michele, Maddaloni (CS). Lonobile T., Baldascino F.

43. A.O.OO.RR.S. Giovanni di Dio e Ruggi d`Aragona - A.O.U. di Salerno, Salerno. Di Benedetto G., Mastrogiovanni G.

44. A.O. San Sebastiano, Caserta. Piazza L., Marmo J.

45. A.O.U. Federico II, Napoli. Vosa C., De Amicis V.

46. Anthea Hospital, Bari. Speziale G., Visicchio G., Spirito R.

47. Casa Di Cura Citta Di Lecce Srl, Lecce. Gregorini R., Specchia L.

48. Azienda Sanitaria Locale Le Fazzi Presidio Ospedaliero Vito Fazzi, Lecce. Villani M., Pano M.A.

49. A.O.U. Consorziale Policlinico di Bari, Bari. Bortone A., De Luca Tupputi Schinosa L., De Cillis E.

50. Azienda Ospedaliera Regionale San Carlo , Potenza. Gaeta R., Di Natale M.

51. S. Anna Hospital, Catanzaro. Cassese M., Antonazzo A.

52. Villa Maria Eleonora Hospital , Palermo. Argano V., Santaniello E.

53. Centro Cuore Morgagni, Pedara (CT). Patanè L., Gentile M., Tribastone S.

54. A.R.N.A.S. Ospedale Civico - Di Cristina - Benfratelli, Palermo. Follis F., Montalbano G.

55. IS.ME.T.T. (Istituto Mediterraneo per i Trapianti e Terapie ad Alta Specializzazione), Palermo. Pilato M., Stringi V.

56. A.O. Ospedali Riuniti Papardo - Piemonte, Messina. Patanè F., Salamone G.

57. A.O.U. Policlinico Paolo Giaccone, Palermo. Ruvolo G., Pisano C.

58. A.O.U. "Policlinico - Vittorio Emanuele" - Ospedale Ferrarotto, Catania. Mignosa C., Bivona A.

59. A.O. Brotzu, Cagliari. Cirio E.M., Lixi G.

OBSERVANT II Investigators:

Coordination

Fulvia Seccareccia, Paola D'Errigo, Stefano Rosato, Gabriella Badoni. National Centre for Global Health - Istituto Superiore di Sanità, Rome, Italy;

Collaborators for the "Ricerca Finalizzata 2016" (PE-2016-02364619)

Corrado Tamburino (PI), Davide Capodanno (Co-PI), Marco Barbanti. A.O.U. Policlinico "G. Rodolico – San Marco" – University of Catania, Catania, Italy

Fausto Biancari. Helsinki University Hospital and University of Helsinki, Helsinki, Finland; Oulu University Hospital, Oulu, Finland

Giovanni Baglio, Francesco Cerza. Agenzia Nazionale per i Servizi Sanitari Regionali (Age.Na.S) – PNE, Rome, Italy

Andrea Marcellusi. Faculty of Economics, University of Rome "Tor Vergata", Rome, Italy

Representatives of the Scientific Societies

- IFC - Italian Federation of Cardiology

Gennaro Santoro. Fondazione "G. Monasterio" CNR/Tuscany Region for the Medical Research and Public Health, Massa, Italy

Gian Paolo Ussia. Campus Bio-Medico University of Rome, Rome, Italy

- GISE – Italian Society of Interventional Cardiology

Giuseppe Musumeci. S. Croce e Carle Hospital, Cuneo

Francesco Bedogni. IRCCS Policlinico S. Donato, S. Donato Milanese, Milan, Italy

Sergio Berti. Fondazione "G. Monasterio" CNR/Tuscany Region for the Medical Research and Public Health, Massa, Italy

Giuseppe Tarantini. University of Padova, Padova, Italy

- ITACTA - Italian Association of Cardiothoracic Anesthesia

Massimo Baiocchi. Policlinico Sant'Orsola, Bologna, Italy

Marco Ranucci. IRCCS Policlinico S. Donato, S. Donato Milanese, Milan, Italy

Institutional collaborations

- National

Domenico Mantoan. Agenzia Nazionale per i Servizi Sanitari Regionali (Age.Na.S), Rome, Italy

- Italian Regional Authorities

Rossana De Palma. Emilia Romagna Region

Salvatore Scondotto. Sicily Region

Anna Orlando. Piemonte Region.

Participating cardiology centers

1. A.O.U. Città della Salute e della Scienza di Torino (TO) - Mauro Rinaldi, Stefano Salizzoni

2. A.O. S. Croce e Carle (CN) - Giuseppe Musumeci, Giorgio Baralis.

3. A.O. SS. Antonio e Biagio e Cesare Arrigo (AL) - Gianfranco Pistis, Maurizio Reale.

4. I.R.C.C.S Policlinico San Donato (San Donato Milanese - MI) - Francesco Bedogni, Giovanni Bianchi.

5. I.R.C.C.S Multimedica (Sesto San Giovanni - MI) - Flavio Airoldi, Iassen Michev.

6. Fondazione I.R.C.C.S. Policlinico San Matteo (PV) - Maurizio Ferrario, Umberto Canosi.

7. ASST Lecco - Ospedale "A. Manzoni" (LC) - Luigi Piatti, Gianluca Tiberti.

8. ASST degli Spedali Civili - Presidio Ospedaliero di Brescia (BS) - Federica Ettori (retired), Salvatore Curello, Marianna Adamo

9. I.R.C.C.S Ospedale San Raffaele (MI) - Antonio Colombo, Matteo Montorfano, Marco Ancona.

10. ASST Monza & Brianza - Ospedale S. Gerardo (MB) - Virgilio Colombo, Ivan Calchera.

11. Fondazione Poliambulanza (BS) - Ornella Leonzi, Diego Maffeo.

12. ASST Papa Giovanni XXIII (BG) - Orazio Valsecchi, Federica Roncali, Angelina Vassileva.

13. Policlinico di Monza (MB) - Filippo Scalise, Giovanni Sorropago.

14. A.O. di Padova - Centro Gallucci (PD) - Giuseppe Tarantini, Alessandro Schiavo.

15. Hesperia Hospital (MO) - Giuseppe D'Anniballe, Davide Gabbieri.

16. A.O.U. di Parma (PR) - Luigi Vignali, Michela Bollettino.

17. A.O.U. Careggi (FI) - Carlo Di Mario, Francesco Meucci.

18. A.O.U. Senese - Ospedale Santa Maria alle Scotte (SI) - Carlo Pierli (retired), Massimo Fineschi, Alessandro Iadanza.

19. Fondazione Toscana Gabriele Monasterio - Ospedale del Cuore "G. Pasquinucci" (MS) - Sergio Berti, Giuseppa Lo Surdo.

20. Ospedale San Filippo Neri (RM) - Giulio Speciale, Andrea Bisciglia.

21. Fondazione Policlinico Universitario Agostino Gemelli IRCCS - Università Cattolica del Sacro Cuore (RM) - Carlo Trani, Diana Verdirosi.

22. A.O. San Camillo Forlanini (RM) - Roberto Violini, Laura Zappavigna.

23. A.O. San Giuseppe Moscati (AV) - Emilio Di Lorenzo, Michele Capasso.

24. A.O.U. Federico II (NA)- Giovanni Esposito, Fabio Magliulo.

25. A.O.U. OO.RR. San Giovanni di Dio e Ruggi d'Aragona (SA) - Pietro Giudice, Tiziana Attisano.

26. A.O.U.C. Policlinico di Bari (BA) - Alessandro Santo Bortone, Emanuela De Cillis.

27. A.O.U. Policlinico-Vittorio Emanuele, Università di Catania (CT) - Corrado Tamburino, Marco Barbanti.

28. Centro Cuore Morgagni - Pedara (CT) - Sebastiano Immè, Martina Patanè.
